# Dissecting the causal effect between gut microbiota, DHA, and urate metabolism: A large-scale bidirectional Mendelian randomization

**DOI:** 10.3389/fimmu.2023.1148591

**Published:** 2023-03-30

**Authors:** Tianzhichao Hou, Huajie Dai, Qi Wang, Yanan Hou, Xiaoyun Zhang, Hong Lin, Shuangyuan Wang, Mian Li, Zhiyun Zhao, Jieli Lu, Yu Xu, Yuhong Chen, Yanyun Gu, Jie Zheng, Tiange Wang, Weiqing Wang, Yufang Bi, Guang Ning, Min Xu

**Affiliations:** ^1^Department of Endocrine and Metabolic Diseases, Shanghai Institute of Endocrine and Metabolic Diseases, Ruijin Hospital, Shanghai Jiao Tong University School of Medicine, Shanghai, China; ^2^Shanghai National Clinical Research Center for Metabolic Diseases, Key Laboratory for Endocrine and Metabolic Diseases of the National Health Commission of the PR China, Shanghai Key Laboratory for Endocrine Tumor, State Key Laboratory of Medical Genomics, Ruijin Hospital, Shanghai Jiao Tong University School of Medicine, Shanghai, China

**Keywords:** gout, uric acid, docosahexaenoic acids, mendelian randomization, mediation, gut microbiome

## Abstract

**Objectives:**

Our aim was to investigate the interactive causal effects between gut microbiota and host urate metabolism and explore the underlying mechanism using genetic methods.

**Methods:**

We extracted summary statistics from the abundance of 211 microbiota taxa from the MiBioGen (N =18,340), 205 microbiota metabolism pathways from the Dutch Microbiome Project (N =7738), gout from the Global Biobank Meta-analysis Initiative (N =1,448,128), urate from CKDGen (N =288,649), and replication datasets from the Global Urate Genetics Consortium (N gout =69,374; N urate =110,347). We used linkage disequilibrium score regression and bidirectional Mendelian randomization (MR) to detect genetic causality between microbiota and gout/urate. Mediation MR and colocalization were performed to investigate potential mediators in the association between microbiota and urate metabolism.

**Results:**

Two taxa had a common causal effect on both gout and urate, whereas the *Victivallaceae* family was replicable. Six taxa were commonly affected by both gout and urate, whereas the *Ruminococcus gnavus group* genus was replicable. Genetic correlation supported significant results in MR. Two microbiota metabolic pathways were commonly affected by gout and urate. Mediation analysis indicated that the *Bifidobacteriales* order and *Bifidobacteriaceae* family had protective effects on urate mediated by increasing docosahexaenoic acid. These two bacteria shared a common causal variant rs182549 with both docosahexaenoic acid and urate, which was located within *MCM6/LCT* locus.

**Conclusions:**

Gut microbiota and host urate metabolism had a bidirectional causal association, implicating the critical role of host-microbiota crosstalk in hyperuricemic patients. Changes in gut microbiota can not only ameliorate host urate metabolism but also become a foreboding indicator of urate metabolic diseases.

## 1 Introduction

Gout is a prevalent inflammatory condition characterized by a sustained high serum urate concentration and intermittent episodes of severely painful arthritis (gout flares) (1). It is the second most common metabolic disease after type 2 diabetes and leads to an increased rate of subsequent cardiovascular complications (2). Epidemiological investigations showed gout prevalence ranging from <1% to 6.8% worldwide, which was highly related to patients’ genetics, lifestyles, and social and economic status (3). Recently, gut microbiota was reported to be associated with the pathogenesis of hyperuricemia. The abundance and composition changes in gut microbiota might increase serum uric acid levels through the dysfunction of uric acid degradation and increased uric acid production (4). Meanwhile, gut microbiota also plays a crucial role in treating metabolic diseases using probiotics and prebiotics (5, 6). Therefore, the human microbiota is gradually recognized as a novel target for a better understanding of the pathogenesis of gout and hyperuricemia.

However, previous studies focusing on the bacteria-urate interplay faced several obstacles, including inadequate participants, cross-sectional design, and less evidence of causality, as well as the fact that most of the studies were conducted within the Asian population (7–10). In addition, the underlying mechanism linking gut bacteria with urate metabolism has not been well established. Gut microbiota and its metabolites were associated with rheumatic diseases including gout, rheumatoid arthritis, and osteoarthritis *via* fatty acids (11). An experimental study investigated the impact of polyunsaturated fatty acids (PUFAs) in the handling of urate by inhibiting urate transporters *in vitro* (12). Some observational studies also suggested that ω-3 PUFAs, especially DHA were highly related to hyperuricemia (13, 14). However, a recent clinical trial found no significant difference in the serum urate level between the 24-week ω-3 PUFAs supplement group and the control group (15). Since gut microbiota might confer an effect on the host health status by metabolizing PUFAs (16), we hereby hypothesize a potential mediation effect of gut microbiota on urate metabolism *via* PUFAs and their important subtypes, such as DHA.

Given the ambiguous connection between gut bacteria and urate metabolism, we used several genetic methods to investigate the bidirectional causal effects between gut microbiota and urate metabolism and further explored potential mediators. Lifelong exposure to genetic methods could draw stable conclusions about the association and provide more evidence for probiotics treatment on urate metabolic disorders.

## 2 Methods

### 2.1 Study design

**Figure 1:**
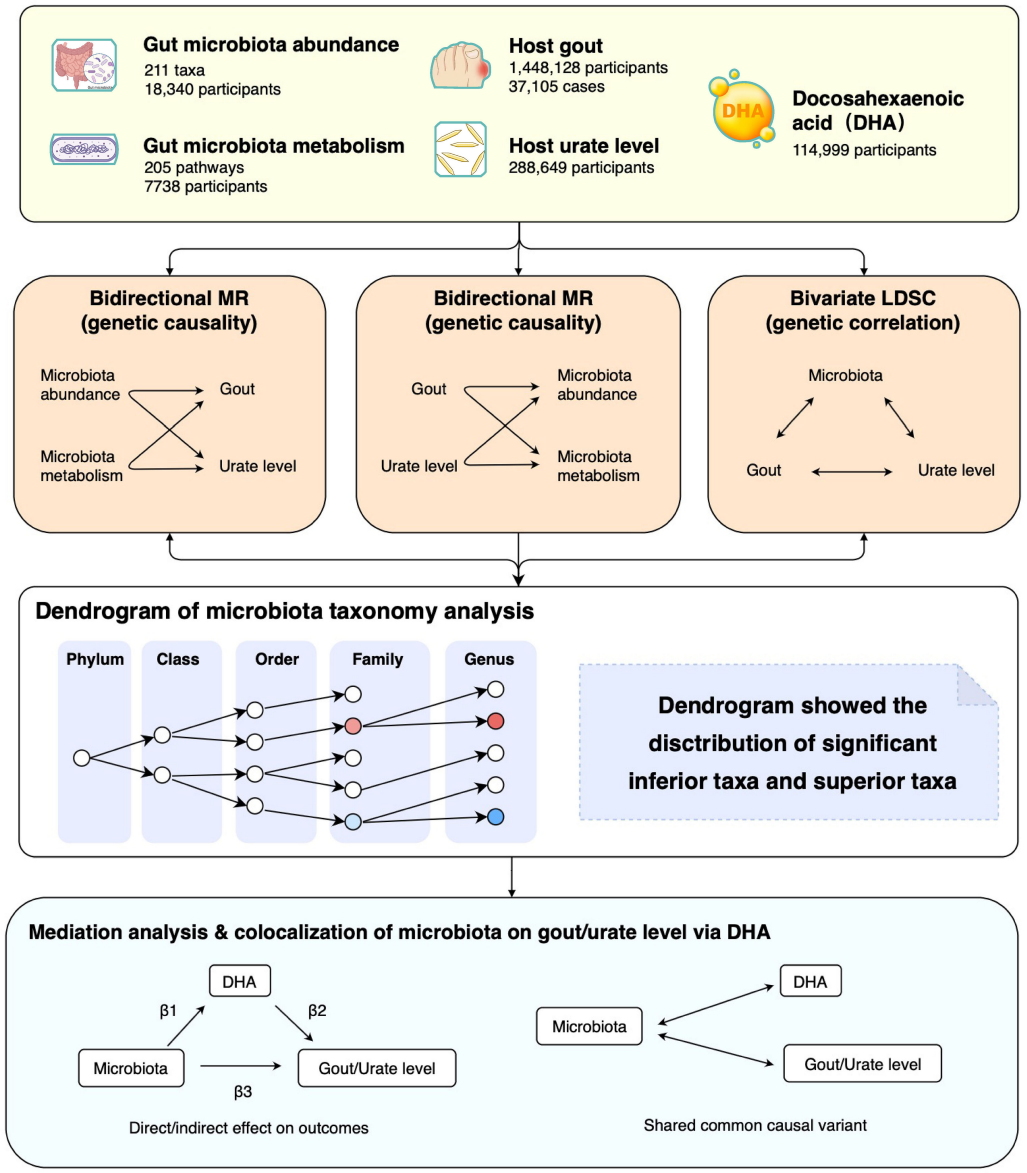
Study design and flowchart. MR, Mendelian randomization; LDSC, Linkage disequilibrium score regression.

Firstly, we extracted summary statistics of gut microbiota, microbiota metabolism pathways, gout, urate level, and docosahexaenoic acid percentage (DHA, a kind of polyunsaturated fatty acid previously mentioned in clinical trials) from the respective consortiums. Secondly, we performed a large-scale bidirectional Mendelian randomization (MR) and bivariate linkage disequilibrium score regression (LDSC) to explore the genetic causality and correlation between microbiota phenotypes (gut microbiota abundance and microbiota metabolism pathways) and urate phenotypes (host gout and urate level). Finally, we used mediation analysis and colocalization to investigate the interaction of gut microbiota, DHA percentage, and urate metabolism (**Figure 1**). The MR analysis was reported as per the STROBE-MR guidelines, and we adopted several methods to follow the three fundamental assumptions of MR (**Table S1**) (17).

### 2.2 Instrument variables selection

Summary statistics for gut microbiota abundance were extracted from a genome-wide association study (GWAS) of host genetic variation in 18,340 multiple-ancestries participants (85% European-ancestry) from the MiBioGen consortium (**Table S2**) (18). It included 211 taxa: 9 phyla, 16 classes, 20 orders, 35 families, and 131 genera determined by the 16S ribosomal RNA gene sequencing. Summary statistics for microbiota metabolism pathways were extracted from the Dutch Microbiome Project (DMP) in 7738 European-ancestry participants, in which the metabolism pathways were determined by shotgun metagenomic sequencing (19). Included in the subsequent analysis were 205 bacteria metabolism pathways.

Summary statistics for gout were from the Global Biobank Meta-analysis Initiative (GBMI) (20). It is a collaborative network of 19 biobanks from four continents representing more than 2.1 million consenting individuals with genetic data linked to electronic health records. We used the gout data of both multi-ancestry (N = 1,448,128, 72% European-ancestry, and N cases =37,105) and European-ancestry (N = 1,069,839; N cases = 30,549). Summary statistics for urate level were from the CKDGen Consortium, including 288,649 European participants (21). We also used phenotypes of both gout (N =69,374 participants) and urate level (N =110,347 participants) from GUGC Consortium as an independent replication analysis (22). Summary statistics for blood DHA and PUFAs were from the largest GWAS for metabolites in the UK Biobank, including 114,999 European participants (23). However, the summary statistics of eicosapentaenoic acid or other specific subtypes of PUFAs were not available for this study.

The IV selection of exposures in bidirectional MR followed the following criteria. For each microbiota taxon and pathway, variants with genome-wide significance *P <*1×10^−5^ and effect allele frequency (EAF) >0.01 were included. All these genetic variants were clumped to a linkage disequilibrium threshold of r^2^ <0.001 using the 1000 Genomes European reference panel. We also calculated the F-statistics to avoid weak instrument bias. Due to the different genotyping platforms of the GWAS, some single nucleotide polymorphisms (SNPs) representing gut microbiota might be missing in the GWAS outcome, which might cause statistical bias if we simply discarded those missing SNPs. Therefore, we used proxy SNPs with LD r^2^ >0.8 as substitutes for these missing SNPs (24, 25). Finally, the remaining genetic variants were used as IVs to model the effect of specific taxa and pathways of gut microbiota. For gout and urate level, SNPs with genome-wide significance *P <*5×10^−8^ and EAF >0.01 were included. All SNPs were clumped to an LD threshold of r^2^ <0.001 using the 1000 Genomes European reference panel (26).

### 2.3 Genetic causality and correlation of microbiota and urate metabolism

We obtained the MR estimates for the causal effect using the inverse-variance weighted (IVW) method (27). The estimate was provided as effect size (β) with a 95% confidence interval (CI). MR results of a specific taxon or pathway which were significant (*P <*0.05) in both gout and urate level with the same direction were defined as “common”. To control type 1 error, a significant taxon (*P <*0.05) that could be replicated in the independent GUGC cohort was defined as “replicated”; other significant MR results (*P <*0.05) were defined as nominally significant. Results of taxa were mapped in the dendrograms to investigate the distribution of significant inferior and superior taxa. The heterogeneity of effects was assessed by Cochran’s *Q* test. We performed several sensitivity analyses using the weighted median, mode-based, MRPRESSO, and contamination mixture methods to validate the results from the IVW method (28–32). We also used the MR-Steiger filtering to determine an actual causal direction. The MR-Egger intercept was used to assess the horizontal pleiotropy. Any results whose *P* value of Egger intercept was <0.05 were excluded.

We also used single-variate LDSC to estimate the heritability of 211 microbiota taxa, gout, and urate level. LD scores were calculated for all high-quality genetic variants (i.e., INFO score > 0.9 and EAF > 0.01) from each GWAS. To further understand the genetic correlation, we conducted a pair-wise genetic correlation analysis of the 211 microbiota taxa and both gout and urate level using bivariate LDSC based on the GWAS summary statistics. The genetic correlation between gout and urate level was also calculated to estimate the genetic similarity in two independent cohorts.

### 2.4 Mediation analysis and colocalization of microbiota, DHA, and gout/urate level

To estimate the effect of DHA acting on gut microbiota, we performed mediation analysis using multivariable MR. Firstly, we conducted an MR of DHA on gout and urate level. Secondly, we performed MR of 19 microbiota (significant effect on gout or urate level) on DHA (β1). Finally, we performed multivariable MR to determine the mediation effect of the DHA in bacteria on gout and urate. The multivariable Mendelian randomization (MVMR) estimated the effect of DHA on gout and urate adjusting for bacteria (β2) and the effect of bacteria on gout and urate adjusting for bacteria. To calculate the indirect mediation effect of bacteria on disease outcomes, we used the product of coefficients method as our primary method, which is the casual effect of bacteria on outcomes *via* DHA (β1×β2). Thus, the proportion of the total effect mediated by DHA was estimated by dividing the indirect effect by the total effect (33, 34).

We then conducted colocalization analysis to test whether the significantly mediated microbiota shared a common causal variant with both DHA and gout/urate level. The posterior probability of the specific variant ( ± 5000 bp) was colocalized with summary statistics of DHA and gout/urate levels.

All MR analyses were based on the TwoSampleMR package in R, version 4.1.2 (24); LDSC was based on LDSC software in Python, version 1.0.1 (35); the colocalization test was performed by the coloc package in R, version 5.1.0.1 (36).

## 3 Results

For 211 taxa in the MiBioGen consortium, the genetic variants used as IVs for each taxon exposure ranged from four to 26 SNPs (median 13 SNPs; F-statistics =21). For 205 metabolism pathways in the DMP, the genetic variants ranged from one to 20 SNPs (median 11 SNPs; F-statistics =25). Additionally, we used 54 SNPs as IVs for gout in GBMI and 88 SNPs for urate level in CKDGen (**Tables S3–S5**).

### 3.1 Bidirectional causal association of gut microbiota and urate metabolism

In the MR analysis, we identified 13 and 9 taxa that causally affected gout and urate level, respectively, whereas two taxa were common in both gout and urate level (**Tables S6, S7**). Conversely, 16 taxa were causally affected by gout and 19 taxa by urate level, whereas 6 taxa were common (**Tables S8, S9**).

**Figure 2:**
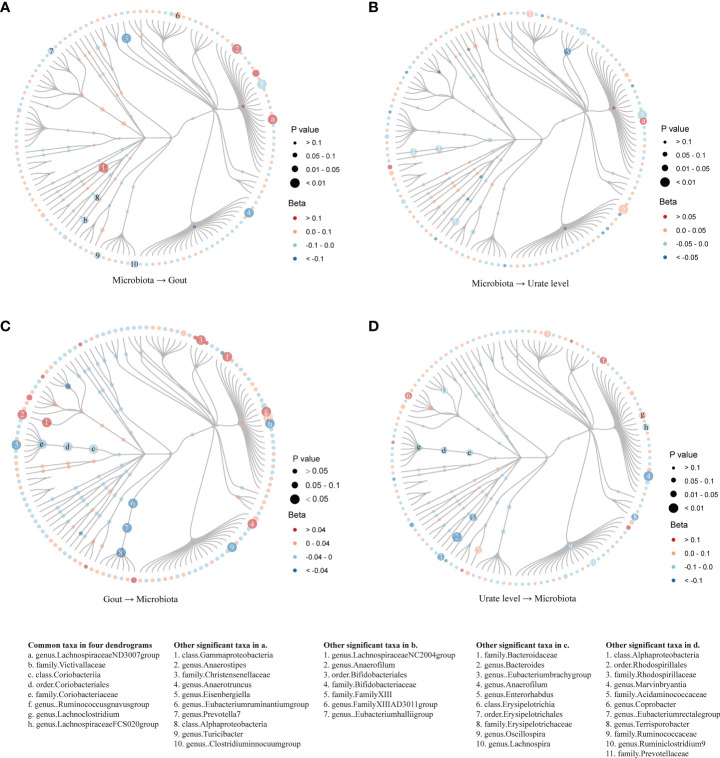
Dendrograms showing the taxonomy structure of the association between gut microbiota and host gout/urate level. Four dendrograms compared with the MR results of inferior and superior taxa according to the taxonomy structure. We included 203 taxa in the figures and excluded eight taxa named unknown. Taxa with significant MR results were listed at the bottom of the figure: the first column showed eight common taxa in both gout and urate level; the other four columns showed significant taxa in the respective figures. **(A)**. MR results from microbiota to gout; **(B)**. MR results from microbiota to urate level; **(C)**. MR results from gout to microbiota; **(D)**. MR results from urate level to microbiota.

In evaluating the causal effect of microbiota on gout and urate level, we found that the increase in abundance of the *Lachnospiraceae ND3007 group* genus commonly had a positive causal effect on both gout and urate level; the *Victivallaceae* family commonly had a negative causal effect on both phenotypes (**Table 1**). The MR-Steiger test supported the direction of significant MR estimates. Other taxa with significant causal effects on gout or urate level are shown in **Figure S1**. We then mapped the MR results of 203 taxa (except for eight unknown taxa) into dendrograms. According to the bacteria taxonomy, **Figures 2A, B** showed that genetically predicted 20 gut microbial taxa had causal effects on urate metabolism. We found that both the *Bifidobacteriaceae* family and its superior *Bifidobacteriales* order had significant causal effects on urate level (since they had the same IVs).

**Table 1:** Bidirectional MR analysis of the causal effect between gut microbiota and gout/urate level.

Taxonomy	Gout				Urate level			
	Effect size β (95% CI)	*P* value	*P* for pleiotropy	Steiger test	Effect size β (95% CI)	*P* value	*P* for pleiotropy	Steiger test
Direction: microbiota → gout/urate level								
*family.Victivallaceae*	-0.04 (-0.08, -0.01)	0.033	0.074	True	-0.02 (-0.04, -0.01)	0.025	0.519	True
*genus.Lachnospiraceae ND3007 group*	0.25 (0.06, 0.43)	0.009	0.942	True	0.09 (0.01, 0.19)	0.044	0.843	True
Direction: gout/urate level → microbiota								
*class.Coriobacteriia*	-0.04 (-0.08, -0.01)	0.039	0.836	True	-0.07 (-0.14, -0.01)	0.025	0.524	True
*order.Coriobacteriales*	-0.04 (-0.08, -0.01)	0.039	0.836	True	-0.07 (-0.14, -0.01)	0.025	0.524	True
*family.Coriobacteriaceae*	-0.04 (-0.08, -0.01)	0.039	0.836	True	-0.07 (-0.14, -0.01)	0.025	0.524	True
*genus.Lachnoclostridium*	0.05 (0.01, 0.09)	0.015	0.725	True	0.07 (0.01, 0.14)	0.028	0.113	True
*genus.Lachnospiraceae FCS020 group*	-0.05 (-0.09, -0.01)	0.038	0.999	True	-0.08 (-0.15, -0.01)	0.039	0.326	True
*genus.Ruminococcus gnavus group*	0.09 (0.02, 0.15)	0.012	0.649	True	0.12 (0.01, 0.23)	0.028	0.275	True

The definition of “common” means a significant (*P* <0.05) result of a specific taxon that could be found in both gout and urate level in the same direction. P for pleiotropy was from the inverse-variance weighted method and meant less pleiotropy in MR analysis if *P* >0.05. The MR Steiger test was used to ensure the right causal direction (not confounded) from microbiota to gout/urate or gout/urate to microbiota.

In evaluating the causal effect of gout and urate level on microbiota, we found that both gout and urate level commonly had a positive causal effect on the *Lachnoclostridium* genus and *Ruminococcus gnavus group* genus; gout and urate level commonly had a negative causal effect on *Coriobacteriia* class, *Coriobacteriales* order, *Coriobacteriaceae* family, and *Lachnospiraceae FCS020 group* genus. The MR-Steiger test supported the direction of significant MR estimates (**Table 1**). Other taxa affected by gout or urate level such as the *Bacteroides* genus, *Lachnoclostridium* genus, and *Eubacterium hallii group* genus can be found in **Tables S8, S9** and **Figure S2**. **Figures 2C, D** show that urate metabolism had a causal effect on 29 gut microbial taxa.

Finally, replicated MR analysis in the independent GUGC cohort further validated our finding. In all common taxa, the effect of the *Victivallaceae* family on both gout and urate level could be replicated in GUGC; the effect of both gout and urate level on the *Ruminococcus gnavus group* genus could also be replicated in GUGC (**Table 2**).

**Table 2:** Replicated significant taxa of bidirectional MR supported the causal effect between gut microbiota and gout/urate level.

Taxonomy	Gout				Urate level			
	Population	Methods	Effect size	P value	Population	Methods	Effect size	P value
Direction: microbiota → gout/urate level								
*Victivallaceae Family*	Primary: Gout from GBMI	IVW	-0.04 (-0.08, -0.01)	0.033	Primary: Urate from CKDGen	IVW	-0.02 (-0.04, -0.01)	0.025
		WM	-0.02 (-0.08, 0.04)	0.492		WM	-0.03 (-0.06, 0.00)	0.057
		MRPRESSO	-0.04 (-0.08, -0.01)	0.040		MRPRESSO	-0.02 (-0.04, -0.01)	0.030
		Con-mix	-0.01 (-0.17, 0.04)	0.697		Con-mix	-0.03 (-0.05, 0.00)	0.052
		Steiger test	True	<0.001		Steiger test	True	<0.001
	Replication: Gout from GUGC	IVW	0.07 (-0.15, 0.29)	0.548	Replication: Urate from GUGC	IVW	-0.05 (-0.09, -0.01)	0.025
		WM	0.09 (-0.19, 0.38)	0.518		WM	-0.05 (-0.10, 0.00)	0.053
		MRPRESSO	0.07 (-0.15, 0.00)	0.567		MRPRESSO	-0.03 (-0.06, 0.00)	0.090
		Con-mix	0.22 (-0.48, 0.62)	0.177		Con-mix	-0.04 (-0.10, -0.01)	0.051
		Steiger test	True	<0.001		Steiger test	True	<0.001
	**Meta-analysis**		**-0.02 (-0.04, -0.01)**	**0.047**	**Meta-analysis**		**-0.01 (-0.02, -0.01)**	**0.003**
Direction: gout/urate level → microbiota								
*Ruminococcus gnavus group Genus*	Primary: Gout from GBMI	IVW	0.09 (0.02, 0.15)	0.012	Primary: Urate from CKDGen	IVW	0.12 (0.01, 0.23)	0.028
		WM	0.07 (-0.02, 0.17)	0.133		WM	0.24 (0.07, 0.41)	0.005
		MRPRESSO	0.09 (0.02, 0.15)	0.016		MRPRESSO	0.08 (0.02, 0.15)	0.016
		Con-mix	0.09 (0.02, 0.18)	0.023		Con-mix	0.12 (0.03, 0.30)	0.021
		Steiger test	True	<0.001		Steiger test	True	<0.001
	Replication: Gout from GUGC	IVW	0.09 (0.02, 0.16)	0.009	Replication: Urate from GUGC	IVW	0.16 (0.05, 0.28)	0.004
		WM	Insufficient SNPs	/		WM	0.15 (0.01, 0.30)	0.039
		MRPRESSO	Insufficient SNPs	/		MRPRESSO	0.16 (0.05, 0.27)	0.010
		Con-mix	0.09 (0.03, 0.18)	0.021		Con-mix	0.20 (0.08, 0.30)	0.005
		Steiger test	True	<0.001		Steiger test	True	<0.001
	**Meta-analysis**		**0.04 (0.02, 0.06)**	**<0.001**	**Meta-analysis**		**0.06 (0.03, 0.09)**	**<0.001**

The MR Steiger test was used to ensure the right causal direction (not confounded) from microbiota to gout/urate or gout/urate to microbiota. The meta-analysis integrated the effect size of IVW methods based on a random-effect model. The bold form showed the results of meta-analysis.

IVW, inversed-variance weighted; WM, weighted median; MRPRESSO, Mendelian Randomization Pleiotropy RESidual Sum and Outlier; Con-mix, contamination mixture; GBMI, Global Biobank Meta-analysis Initiative; GUGC, Global Urate Genetics Consortium.

Bivariate LDSC supported the genetic correlation of the *Eubacteriumruminantium -group* genus with gout (regression coefficient [Rg]: 0.28, *P* = 0.041) and the *Lachnoclostridium* genus with urate level (Rg: 0.28, *P* = 0.008), which were consistent with the MR results. Gout and urate level were highly genetically correlated (Rg: 0.89, *P <*0.001) (**Tables S10, S11**).

**Figure 3:**
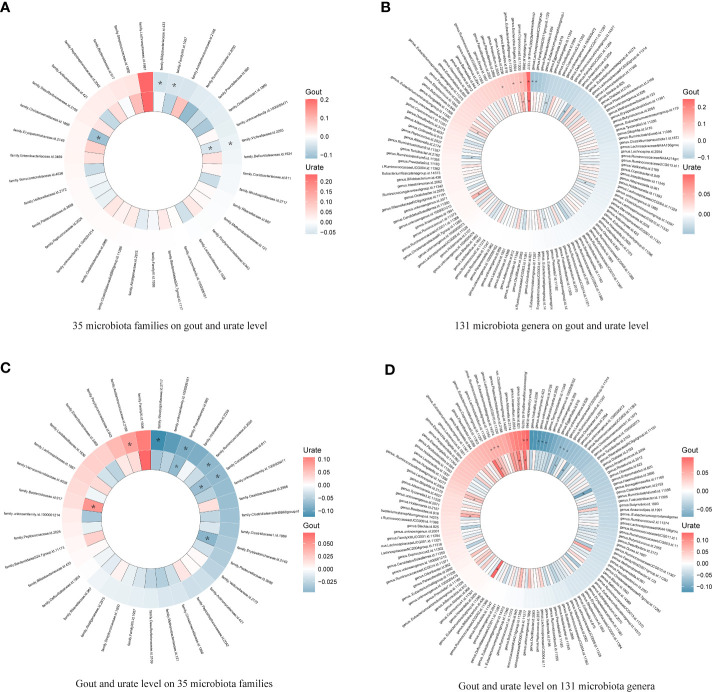
Heatmap showing bidirectional causality of 35 families and 131 genera. Four heatmaps comparing the results of gout with the urate level of each taxon in 35 families and 131 genera. The outer ring represents urate level and the inner ring represents gout. **(A)**. 35 microbiota families on gout and urate level; **(B)**. 131 microbiota genera on gout and urate level; **(C)**. Gout and urate level on 35 microbiota families; **(D)**. Gout and urate level on 131 microbiota genera. * means significant results.

Since few significant taxa appeared in the phylum, class, and order level, we further compared the results of gout with the urate level of each taxon in the family and genus level (**Figure 3**). In the genus level, five genera (one was common) in the *Lachnospiraceae* family had a significant causal effect on either gout or urate level; gout or urate level had a significant causal effect on five genera in the *Lachnospiraceae* family (two were common) (**Figure S3**). Totally, nine genera of the *Lachnospiraceae* family had a significant causal association with gout or urate level, whereas the *Lachnospiraceae NC2004 group* genus could significantly affect host urate level and be affected by host urate level.

### 3.2 Bidirectional causal association of microbiota metabolism pathways and urate metabolism

**Figure 4:**
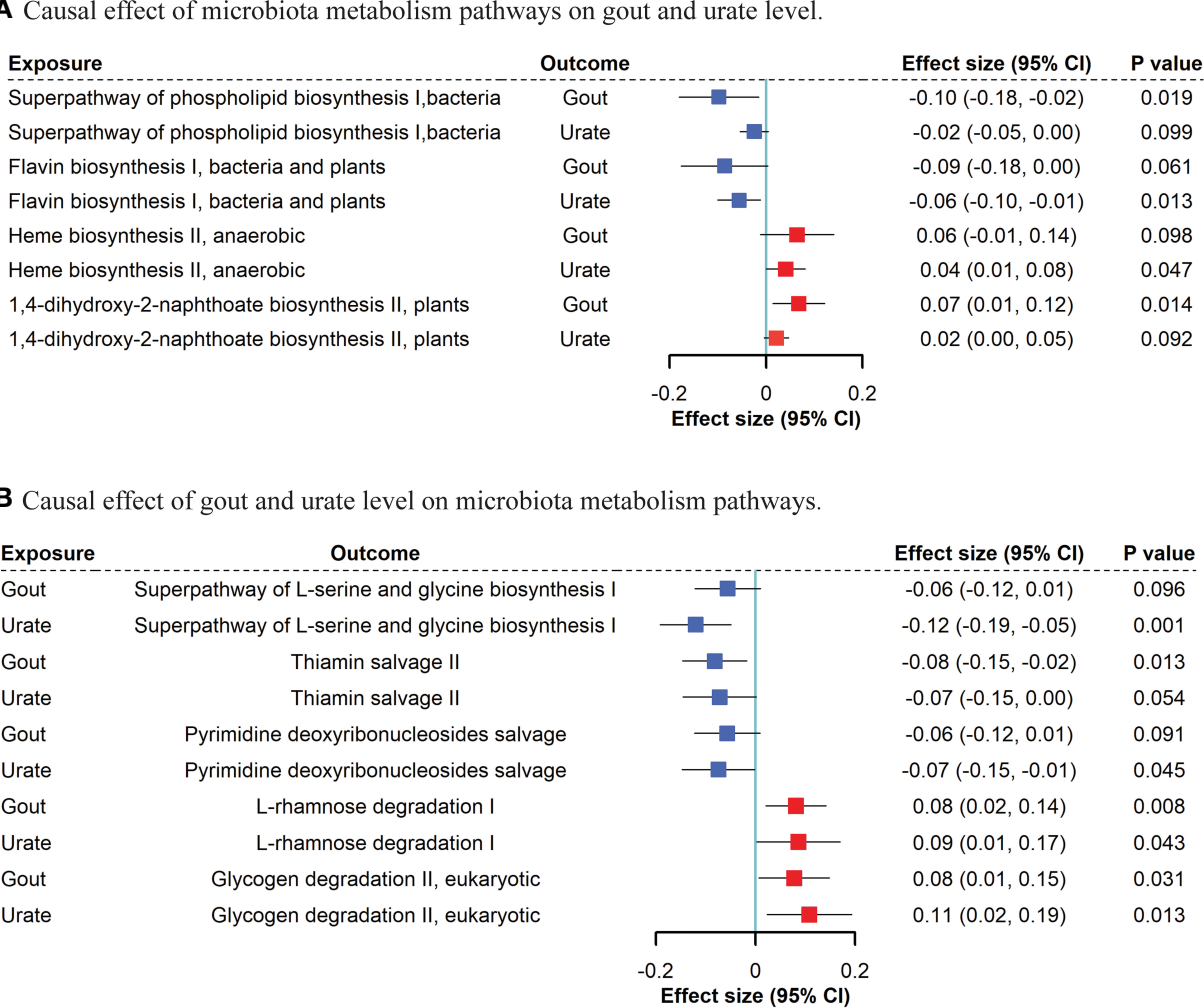
Bidirectional MR suggestive causal association between microbiota metabolism pathways and gout/urate level. **(A)**. Causal effect of microbiota metabolism pathways on gout and urate level. **(B)**. Causal effect of gout and urate level on microbiota metabolism pathways. We included “suggestive” significant pathways in **Figure 4** (*P <*0.05 in gout or urate level and *P <*0.1 with the same direction in the other phenotype).

Based on the MR results (**Tables S12, S13**), we identified that a per unit increased abundance of eight pathways had causal effects on gout and 14 pathways had causal effects on urate level, including 1,4-dihydroxy-2-naphthoate biosynthesis (gout: β: 0.07, 95% CI: [0.01, 0.12], *P* = 0.014) or flavin biosynthesis (urate level: β: -0.06, 95% CI: [-0.10, -0.01], *P* = 0.013) (**Figure 4**).

For gout and urate level on microbiota, we found that both gout presence and urate level had a causal effect on bacterial L-rhamnose degradation (gout: β: 0.08, 95% CI: [0.02, 0.14], *P* = 0.008; urate level: β: 0.09, 95% CI: [0.01, 0.17], *P* = 0.043) and glycogen degradation (gout: β: 0.08, 95% CI: [0.01, 0.15], *P* = 0.031; urate level: β: 0.11, 95% CI: [0.02, 0.19], *P* = 0.013). Other five bacterial metabolism pathways affected by gout and nine pathways affected by urate level can be found in **Tables S14, S15**.

Among all 1654 analyses, 80 non-significant results with Egger intercept *P <*0.05 were excluded. Little pleiotropy remained in the remaining significant results (**Table S16**). Among all 3318 analyses, 196 non-significant IVW results with Cochrane’s Q test *P <*0.05 were excluded. Little heterogeneity remained in the significant results (**Table S17**).

### 3.3 Mediation analysis of microbiota, DHA, and gout/urate level

Firstly, DHA had a significant causal effect on both gout (β: -0.09, 95% CI: [-0.17, -0.01], *P* =0.021) and urate level (β: -0.07, 95% CI: [-0.11, -0.03], *P <*0.001). Secondly, given the protected effect of DHA, among 19 taxa causally associated with gout or urate level, *Bifidobacteriales* order and *Bifidobacteriaceae* family (both β: 0.06, 95% CI: [0.01, 0.13], *P* =0.044) were positively associated with DHA level and negatively associated with urate level (β: -0.06, 95% CI: [-0.11, -0.01], *P* =0.020) (**Table S18**). In the mediation analysis, the indirect effect of *Bifidobacteriales* order on urate level *via* DHA was (β: -0.004, 95% CI: [-0.003, -0.01]) and the proportion of DHA mediation was 7.6% (*P* =0.016). Consistent with DHA, PUFAs level also had a causal effect on both gout (β: -0.40, 95% CI: [-0.62, -0.18], *P <*0.001) and urate level (β: -0.21, 95% CI: [-0.29, -0.12], *P <*0.001), while the *Bifidobacteriales* order *and Bifidobacteriaceae* family had a significant causal effect on serum PUFAs level (both β: 0.04, 95% CI: [0.01, 0.08], *P* =0.010) (**Table S19**). However, the indirect effect of the *Bifidobacteriales* order on urate level *via* PUFAs was not statistically significant (β: -0.0097, 95% CI: [-0.020, 0.034], *P* =0.667).

We then estimated the causal effect of the *Bifidobacteriales* order and *Bifidobacteriaceae* family on urate level under a stricter significance threshold of IVs (*P <*1×10^-8^). The Wald ratio showed the only remaining rs182549 (*P* =5.9×10^-20^) had a significant causal effect on DHA level (β: 0.22, 95% CI: [0.15, 0.30], *P <*0.001). We then accessed the colocalization evidence to the 5000 bp region encompassing the SNP rs182549 at *MCM6* locus using the summary statistics from *Bifidobacteriales* order and *Bifidobacteriaceae* family, DHA, and urate level. The colocalization posterior probability (H4) of COLOC is high with 0.999 in both DHA and urate, which indicated the *Bifidobacteriales* order and *Bifidobacteriaceae* family shared a specific common variant rs182549 with both DHA and urate. We then searched the PhenoScanner database for rs182549 and mapped this variant in the *MCM6* gene combined with *LCT* in the pQTL analysis.

## 4 Discussion

In the present study, we found that 20 genetically predicted taxa significantly affected urate metabolism, and 29 taxa were affected by urate metabolism, followed by supportive genetic correlation from LDSC. Two taxa had a common causal effect on both gout and urate, whereas the *Victivallaceae* family was replicable in the independent GUGC cohort. Six taxa were commonly affected by both gout and urate, whereas the *Ruminococcus gnavus group* genus was replicable in the GUGC. DHA may mediate the protective effect of *Bifidobacteriales* order and *Bifidobacteriaceae* family on the host urate level. *Bifidobacteriales* order and *Bifidobacteriaceae* family shared a common causal variant rs182549 associated with *MCM6/LCT* with both DHA and urate level. To our knowledge, this is the first study to comprehensively examine the genetic association between gut microbiota and urate metabolism. Our findings implicated the critical role of gut microorganisms in host-microbiota crosstalk of urate metabolism disorders, underlying the importance of modulating host-microbe balance in the prevention and treatment of hyperuricemia diseases.

Gut microbiota plays an essential role in the production, catabolism, metabolism, and excretion of host uric acid. It could either convert purines to uric acid by secreting active enzymes or accelerate uric acid degradation by synthesizing urate-metabolizing enzymes (37). However, most studies focusing on the interaction of gut microbiota and uric acid metabolism were cross-sectional studies, which could hardly determine the chronological order of the change in uric acid level and microbiota abundance (7–10). For example, elevated serum urate level could be related to the change in abundance of both purine-decomposition and purine-synthesis bacteria (5). Whether this change in abundance is pathogenic or compensatory for hyperuricemia remains unclear. The present study used bidirectional MR and MR Steiger direction tests to ensure the right causal direction between microbiota and urate metabolism (38). We found that the *Victivallaceae* family and *Lachnospiraceae ND3007 group* genus had a causal effect on both gout and urate level. The former taxon has not been well investigated, while the latter taxon belonged to an important disease-inducing family, the *Lachnospiraceae* family (39). Our MR results indicated five genera in this family had causal effects on gout or urate metabolism. However, the negative results of other taxa in the *Lachnospiraceae* family were not insignificant. Due to the controversial role of different inferior genera, function-dependent cluster analysis or taxon-to-taxon ratio on urate metabolism is warranted for further investigation (39).

In turn, gout and hyperuricemia could also trigger variations in gut microbiota abundance and metabolism. Consistent with observatory studies, the abundance of the *Coriobacteriaceae* family, *Prevotellaceae* family, *Lachnoclostridium* genus, and *Bacteroides* genus changed in patients with gout during treatment (9, 40–42). Therefore, similar to biomarkers of specific diseases, microbial dysbiosis and metabolic disorders serve as a profound reference for future studies in uric metabolism. Patients’ fecal microbiome could be considered a pre-diagnostic target of gout and hyperuricemia (43).

Our results highlighted the different profiles of significant taxa in gout and urate level. Hyperuricemia and gout share common characteristics but also have differences. While a large proportion of individuals with hyperuricemia have never had a gout flare, some patients with gout can have a normuricemia status (44, 45). Hyperuricemia is generally considered to be the pathophysiologic basis of gout flares, and uric acid has dual effects *in vivo* with antioxidant properties as well as being an inflammatory promoter, which places it in a delicate position in balancing metabolisms (46). On the other hand, gout is a multifactorial metabolic disease, and its pathogenesis should not rely solely on hyperuricemia or monosodium urate crystals (46). Thus, the spectrum of significant taxa, combined with the mediation effect of DHA of hyperuricemia was quite different from that of gout; this might explain the difference between gout and urate metabolism in the MR analysis.

*Bifidobacteria* is a well-known probiotic to treat hyperuricemia, which is consistent with our MR (47). Studies indicated that *Bifidobacteria* supplement could elevate DHA levels in animal experiments and DHA supplement could improve hyperuricemia in humans (12, 48). We found that both the *Bifidobacteriales* order and *Bifidobacteriaceae* family had a protective effect on urate metabolism partially through DHA level, whereas rs182549 in *MCM6* was the most predominant variant. *MCM6* could be activated by both DHA supplements in the human body and hyperuricemia status in mice (49, 50). The complex *MCM6/LCT* variation also participated in several diseases’ progress such as obesity, lactose intolerance, and irritable bowel syndrome (51, 52). This suggested that rs182549, which is related to *Bifidobacteria*, might play a critical role in the interplay of gut microbiota, unsaturated fatty acid, and urate metabolism. Furthermore, *MCM6/LCT* might be a potential therapeutic target for treating hyperuricemia.

Although gut microbiota was previously thought to be influenced by host health status, the GWAS significant variants representing the microbiota phenotype were relatively not largely confounded (19). Also, we assessed the genetic effect of microbiota in both gout and urate level. These two statistics were highly correlated but were derived from two independent populations. This not only avoided sample overlap but also replicated MR results in a related phenotype.

This study has several limitations. Firstly, a relatively non-significant heritability of the gut microbiota of the MiBioGen and DMP GWAS might impair the detective power of MR. However, owing to the sufficient sample size of publicly available data from the largest GWAS such as MiBioGen, DMP, and GBMI, the weak instrument bias could be somewhat complemented. Thus, we had adequate power to detect significant lifelong causality. Secondly, due to the difference between gout and urate, it is predictable that some bacteria related to one phenotype could not be replicable in the other phenotype. Therefore, some significant taxa in gout might not be associated with the urate level but might be associated more with systematic inflammation and intestinal transportation (53, 54). This could partly explain the different significant taxa in the two phenotypes.

In conclusion, this is the first study comprehensively assessing the association between gut microbiota and urate metabolism based on genetic methods. Our findings underlined the critical role of gut microorganisms in host hyperuricemia pathogenesis and progress. Taking advantage of host-microbiota crosstalk in urate metabolism could not only indicate directions of clinical prediction and the monitoring of gout but also draw a future blueprint for a cutting-edge therapeutic method based on fecal bacteria transplantation.

## Data availability statement

The original contributions presented in the study are included in the article/**Supplementary Material**. Further inquiries can be directed to the corresponding author.

## Author contributions

MX, TH, YB and GN conceived and designed the study. TH, QW, HD and YH conducted the analysis and finished writing the paper. XZ, JZ, TW, ML, HL, SW, ZZ, YG and YX offered guidance and methods for the analysis and data selection. YC, JL, WW, GN and YB reviewed the article and offered clinical advice. MX, YB and GN took responsibility for the contents of the article. All authors contributed to the article and approved the submitted version.
