# Supplementary material for: Dissecting the causal effect between gut microbiota, DHA, and urate metabolism: A large-scale bidirectional Mendelian randomization
Source: Front Immunol. 2023 Mar 30;14:1148591. doi: 10.3389/fimmu.2023.1148591 (PMC10097983; doi:10.3389/fimmu.2023.1148591)

Figure S1

A. Significant results of taxa on gout (P <0.05).

Exposure	Outcome	Effect size (95% CI)	P value
family.Christensenellaceae	Gout	-0.11 (-0.18, -0.03)	0.005
genus.Anaerotruncus	Gout	-0.11 (-0.18, -0.03)	0.006
class.Alphaproteobacteria	Gout	-0.09 (-0.17, -0.01)	0.022
genus.Turicibacter	Gout	-0.08 (-0.16, -0.01)	0.045
genus.Eisenbergiella	Gout	-0.07 (-0.12, -0.02)	0.007
genus..Clostridiuminnocuumgroup	Gout	-0.06 (-0.12, 0.00)	0.041
genus.Prevotella7	Gout	-0.05 (-0.09, -0.01)	0.042
family.Victivallaceae	Gout	-0.04 (-0.08, -0.01)	0.033
genus..Eubacteriumruminantiumgroup	Gout	0.07 (0.00, 0.13)	0.035
genus.Coprococcus3	Gout	0.12 (0.01, 0.24)	0.039
genus.Anaerostipes	Gout	0.13 (0.04, 0.22)	0.006
class.Gammaproteobacteria	Gout	0.15 (0.04, 0.26)	0.009
genus.LachnospiraceaeND3007group	Gout	0.25 (0.06, 0.43)	0.009

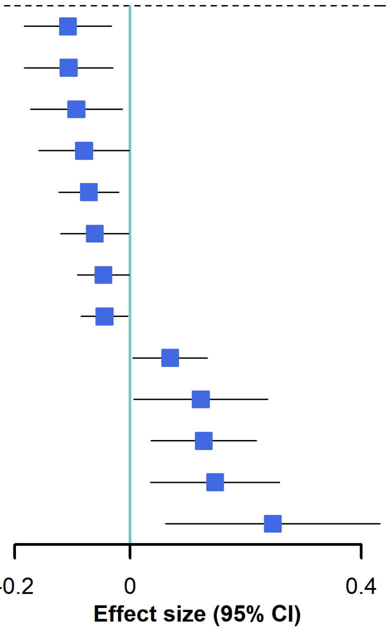

B. Significant results of taxa on urate level (P <0.05).

Exposure	Outcome	Effect size (95% CI)	P value
order.Bifidobacteriales	Urate	-0.06 (-0.11, -0.01)	0.019
family.Bifidobacteriaceae	Urate	-0.06 (-0.11, -0.01)	0.019
family.FamilyXIII	Urate	-0.05 (-0.1, -0.01)	0.024
genus.LachnospiraceaeNC2004group	Urate	-0.05 (-0.08, -0.02)	0.004
genus.FamilyXIIIAD3011group	Urate	-0.05 (-0.09, -0.01)	0.032
family.Victivallaceae	Urate	-0.02 (-0.04, -0.01)	0.025
genus.Anaerofilum	Urate	0.03 (0.01, 0.06)	0.008
genus..Eubacteriumhalliigroup	Urate	0.04 (0.01, 0.08)	0.048
genus.LachnospiraceaeND3007group	Urate	0.10 (0.01, 0.19)	0.044

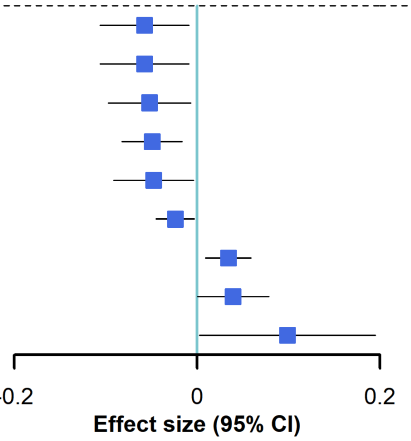

Supplement: Supplementary file 1 [file DataSheet_1.pdf]
