# Supplementary figures and images for: Dissecting the causal effect between gut microbiota, DHA, and urate metabolism: A large-scale bidirectional Mendelian randomization
Source: Front Immunol. 2023 Mar 30;14:1148591. doi: 10.3389/fimmu.2023.1148591 (PMC10097983; doi:10.3389/fimmu.2023.1148591)

Figure S2

A. Significant results of gout on taxa ( $P < 0.05$ ).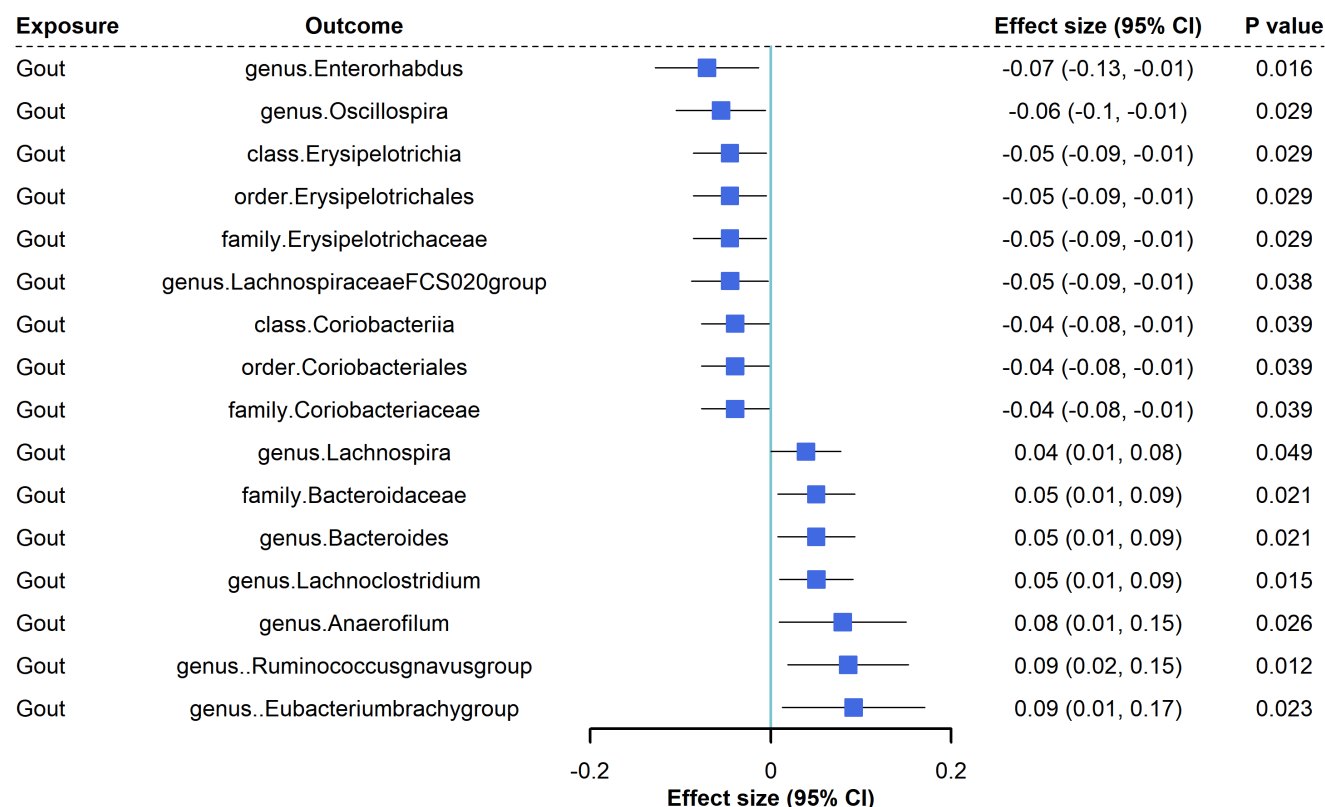B. Significant results of urate level on taxa ( $P < 0.05$ ).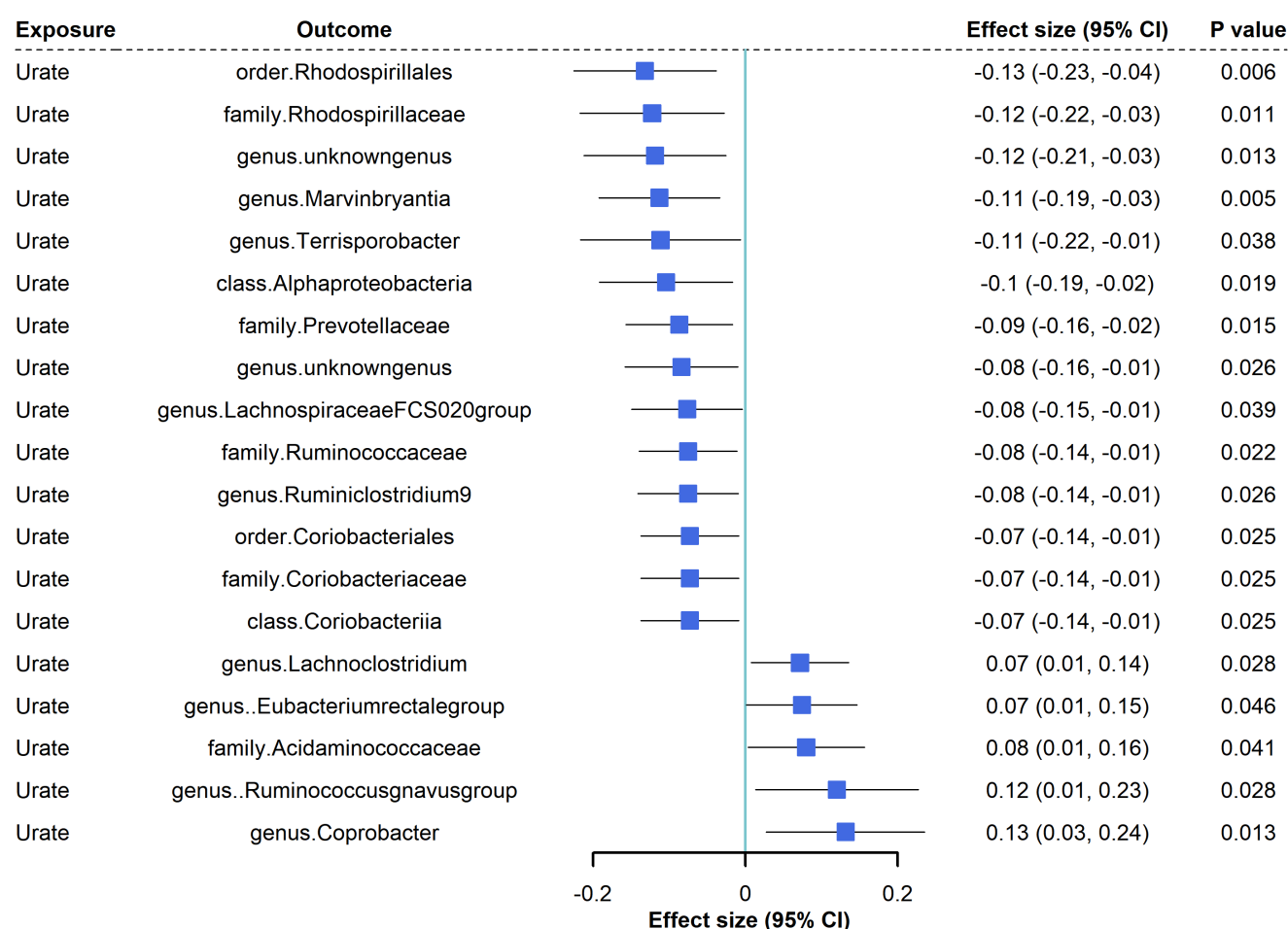

Supplement: Supplementary file 2 [file DataSheet_2.pdf]

Figure S3

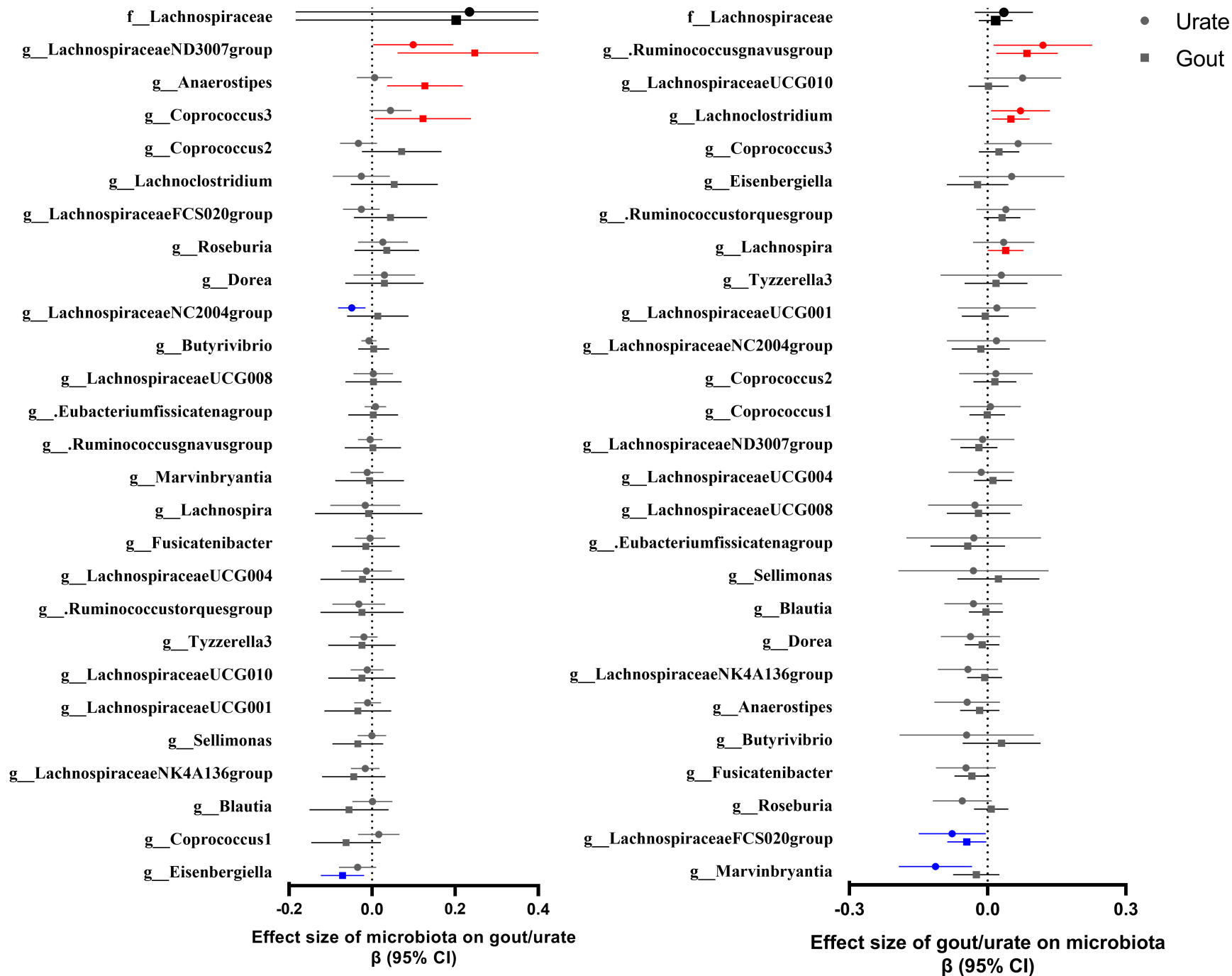

Supplement: Supplementary file 3 [file DataSheet_3.pdf]
